# Epithelial ovarian cancer-secreted exosomal miR-222-3p induces polarization of tumor-associated macrophages

**DOI:** 10.18632/oncotarget.9246

**Published:** 2016-05-09

**Authors:** Xiang Ying, Quanfeng Wu, Xiaoli Wu, Qinyi Zhu, Xinjing Wang, Lu Jiang, Xin Chen, Xipeng Wang

**Affiliations:** ^1^ Department of Gynecology, Shanghai First Maternity and Infant Hospital, Tongji University School of Medicine, Shanghai, China

**Keywords:** exosomes, miR-222-3p, macrophage, polarization, epithelial ovarian cancer

## Abstract

Cancer secreted exosomal miRNAs are emerging as mediators between tumor-stoma crosstalk. Here, we show epithelial ovarian cancer (EOC)-derived exosomes activated macrophages to a tumor-associated macrophage (TAM)-like phenotype with SOCS3/STAT3 pathway involvement, which could facilitate the progression of cancer. MiR-222-3p was enrichment in exosomes released from EOC cells and it could be transferred to macrophages. Overexpression of miR-222-3p in macrophages induced polarization of the M2 phenotype. Luciferase assay verified miR-222-3p targeted SOCS3 genes and expression of SOCS3 was decreased after transfection with a miR-222-3p mimic. Down-regulation of SOCS3 correlated with an increased expression of STAT3 activation. MiR-222-3p could be detected in the exosomes from serum and its levels were related to EOC. These observations propose tumor-derived exosomal miR-222-3p is an effective regulator in the polarization of tumor-promoting M2 macrophages and may be a biomarker of EOC.

## INTRODUCTION

Epithelial ovarian cancer (EOC) represents the fifth most common cause of cancer death in women [1]. Most EOC patients are diagnosed at an advanced stage (FIGO III and IV) [2]. Therefore, elucidating the molecular mechanism in EOC development is essential to develop an early diagnosis and novel target therapy.

There is growing recognition cancer should be considered a complex microenvironment that plays a significant role in tumor progression and metastasis, rather than as single tumor cells [3]. Macrophages displaying diverse phenotypes and functions [4, 5] represent the largest number of immune-related stromal cells in the tumor environment [6]. Our previous study suggested Thrombin and coagulation factor XII could modulate the differentiation of monocytes toward tumor-associated macrophages that facilitate the peritoneal metastasis of EOC [7, 8]. Stimulation with T helper (Th) 1 and Th2 can activate macrophage phenotypes either classically activated macrophages (M1) or alternatively activated macrophages (M2) [9]. Tumor-associated macrophages (TAMs) infiltrate tumors are generally considered to more closely resemble M2 phenotypes [10]. However, the mechanisms result in the changing phenotypes of macrophages remain to be explored.

Exosomes, which are tiny vesicles 30 to 150 nm in size that are formed during endocytosis [11, 12], have been reported to play an essential role in intercellular communication between tumor and stromal cells through transferring their genetic contents. Recently, several studies have shown exosomes from diversified types of cells play important roles in immune modulation [13]. For example, DCs induced CD8 T cell-dependent anti-tumor effects both in mice and in patients with malignant gliomas treated with tumor-derived exosomes [14]. Exosomes derived from tumor promoted T-cell dysfunction and progression in human nasopharyngeal carcinoma [15]. In ovarian cancer, exosomes derived from ascites of the patients could induce apoptosis of the precursors of DCs, DCs and PBMCs [16]. Szajnik et al. showed plasma-derived exosomes of ovarian cancer patients promoted function of Treg, which may support immune evasion of cancers [17].

Activation of JAK/STAT pathway plays a crucial roles in differentiation and polarization of macrophages. SOCS3 is a negative feedback regulator of the JAK/STAT signaling pathway [18], which has been indicated to regulate polarization of M1 and M2 macrophages [19–21]. TargetScan predicted the 3′ UTR of SOCS3 was potentially targeted by miR-222-3p. MiR-222 is over expressed in diverse tumor types, including ovarian cancer [22, 23]. Moreover, our study proved exosomes from serum of EOC express higher levels of miR-222-3p.

In this study, the phenomenon of EOC-derived exosomes inducing macrophages to M2-like polarization was observed. What's more, the underlying mechanisms that could potentially aid in the treatment and detection of EOC were defined.

## RESULTS

### Identification of exosomes derived from EOC cells and uptake of the exosomes in macrophages

**Figure 1:**
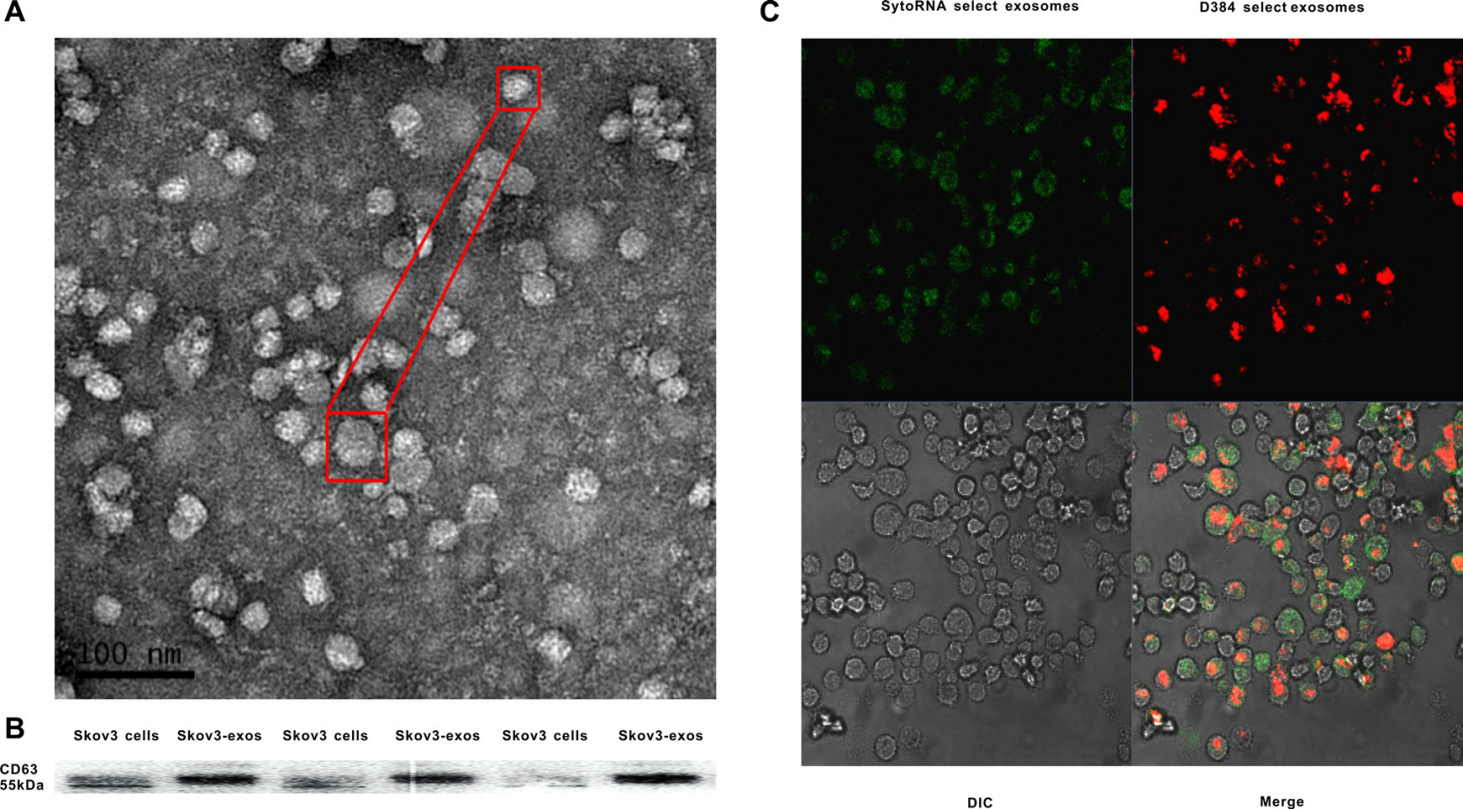
Identification of Skov3 secreted exosomes and internalization of the exosomes into recipient cells (**A**) Representative TEM micrograph of exosomes derived from Skov3 cells. (**B**) Immunoblot of CD63 in exosomes derived from Skov3 cells. (**C**) Unlabeled macrophages were incubated with Skov3-derived exosomes that were labeled with D384 and SytoRNA. The differential interference contrast (DIC) presented the morphology of macrophages which were not fluorescent. Data exhibits typical image of 3 independent experiments.

Exosomes extracted from the culture supernatant of Skov3 cells were isolated using a total exosome isolation reagent. Transmission electron microscopy (TEM) revealed the morphology and size of the acquired exosomes. The size ranged from 30 to 80 nm and had a cup-shape appearance (Figure 1A). The characterization of the exosomes was confirmed by the presence of known exosomal marker CD63, a member of tetraspanin family proteins using western blot (Figure 1B). Because exosomes are rich in RNA molecules [24], we next explored whether exosomes could be taken up by recipient cells prior to transferring their contents. Exosomes with fluorescence were labeled and then cultured with unstained macrophages. Examination with a confocal microscope revealed the macrophage cytoplasm was stained (Figure 1C).

### EOC-derived exosomes activate macrophages to a TAM-like phenotype *in vitro* and *vivo*, which can promote growth and metastasis of EOC

**Figure 2:**
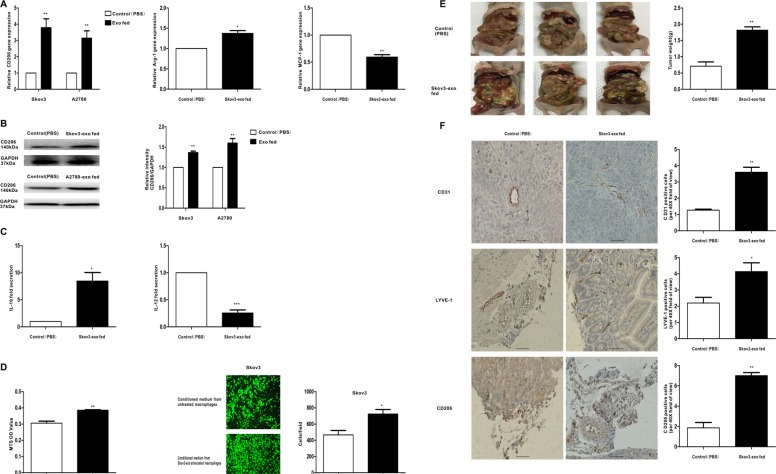
Exosomes derived EOC cells activate macrophages to a TAM-like phenotype *in vitro* and *vivo*, which can promote the progression of EOC Macrophages were treated with EOC-derived exosomes (100 ug/ml) or control (PBS). (**A**) After 48 hours, qRT-PCR was applied using primers for CD206, Arg-1, or MCP-1. (**B**) After 96 hours, the expression of M2 surface marker CD206 was detected by western blot. (**C**) Cytokine levels in the media of macrophages. (**D**) Proliferation and migration capacity of Skov-3 human ovarian cancer cells which incubated with the supernatants of macrophages treated with the exosomes or control (PBS) was performed using MTS and Transwell assay. Cell viability was determined by OD measurement and migrated cells were counted. (**E**) Photographs of appearance of tumor deposits at necropsy of the injected mice. (**F**) Representative IHC examples of CD31, LYVE-1 and CD206 staining are shown in tumors of Skov3-exo group and compared with control group; scale bar = 50 μm. CD31 positive ‘hotspots’, LYVE-1 positive ‘hotspots’ and CD206-positive cells were calculated as number of positive cells. Data are shown as mean ± SEM, *n* = 3 independent experiments; *p < 0.05, **p < 0.01, and ***p < 0.001 compared with the control treatments.

To determine whether exosomes derived from EOC cells have the ability to promote the polarization of the M2 phenotype of macrophages, macrophages were treated with exosomes extracted from Skov3 and A2780 cells (100 μg/ml). Then, the expression of M1 and M2 typical markers was detected. Compared with controls, CD206 was rose up when treated with the Skov3-derived and A2780-derived exosomes (Figure 2A and Figure 2B). Besides, the expression of Arg-1 showed increased trends whereas the expression of MCP-1 showed decreased trends resulting from Skov3-derived exosomes stimulation (Figure 2A). M1 macrophages produce high level IL-12 whereas M2 macrophages secret IL-10 [25]. Consistent with the changes in surface markers, macrophages treated with Skov3-derived exosomes produced a higher level of IL-10 and a lower level of IL-12 (Figure 2C). Furthermore, we explored whether macrophages educated by EOC-derived exosomes could promote proliferation the migration capacity of the EOC cells *in vitro* system. The human EOC cell line Skov3 was used, and MTS assay showed treatment with the conditioned medium from Skov3-derived exosomes-stimulated macrophages increased viability in EOC cells (Figure 2D). Transwell migration assays indicated the number of migrating EOC cells significantly increased when incubated with the conditioned medium from Skov3-derived exosomes-treated macrophages (Figure 2D), thus illustrating that EOC-derived exosomes-treated macrophages have promoted the proliferation and migratory properties of EOC cells.

After confirming the role of EOC-derived exosomes in regulating the phenotype of macrophages *in vitro*, we examined its function *in vivo*. Skov3 cells mixed with Skov3-exo stimulated or PBS treated macrophages were injected intraperitoneally into the mice. Compared with the control group, the size and number of tumors in the Skov3-exo group were significantly increased (Figure 2E). Additionally, the number of CD206-positive cells in tumor tissue was increased in the Skov3-exo group (Figure 2F, upper 3 panels). Furthermore, the Skov3-exo group showed increase in microvessel and lymphatic vessel density relative to the control (Figure 2F). Collectively, these results suggest EOC-derived exosomes increased polarization of M2 macrophages and promoted angiogenesis and lymphangiogenesis in tumor microenvironment, which accelerated progression of EOC.

### EOC-derived exosomes down-regulate the expression of SOCS3 and activate SOCS3/STAT3 signaling pathway in macrophages

**Figure 3:**
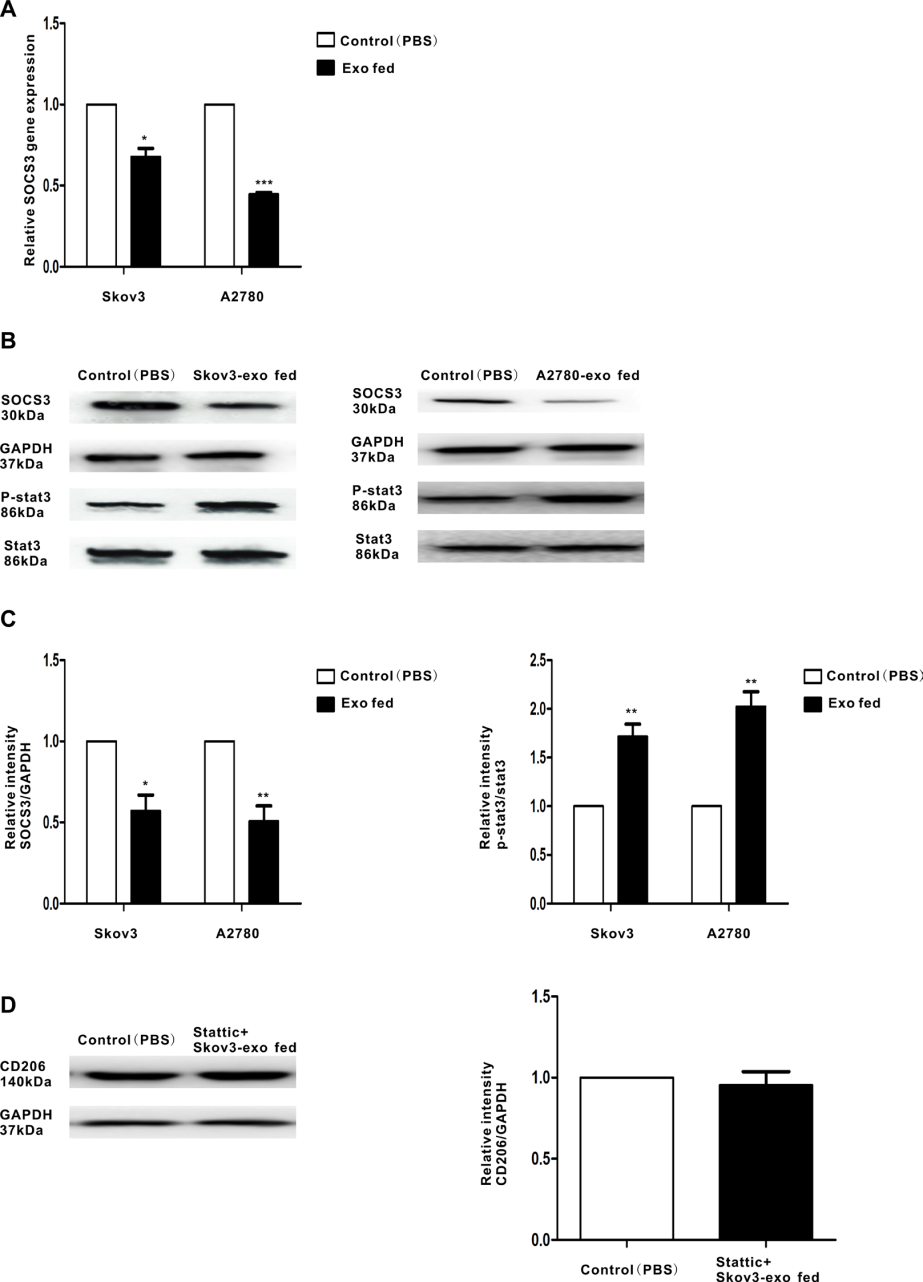
Regulation of SOCS3 expression and the related signaling pathway by EOC-derived exosomes Macrophages were treated with EOC-derived exosomes (100 ug/ml) or control (PBS). (**A**) Expression of SOCS3 gene in macrophages that were stimulated with EOC-derived exosomes or control (PBS), as performed by qRT-PCR. (**B**) A representative immunoblot of SOCS3, phosphorylated (p-) STAT3 and total STAT3 in macrophages which were treated with EOC-derived exosomes or control (PBS) is displayed along with (**C**) quantitative data by densitometry. (**D**) Macrophages were treated with phosphor-STAT3 inhibitor stattic prior to stimulate with Skov3-derived exosomes. Expression of M2 surface marker CD206 was detected by western blot. Data are shown as mean ± SEM, *n* = 3 independent experiments.*p < 0.05 and **p < 0.01 compared with the control treatments.

Activation of the STAT3 signaling pathway participates in M2 polarization, which promotes tumor progression [26, 27]. Over activated STAT3 pathway was related to the immunosuppressive activities of TAMs [28]. Thus, we speculated EOC-derived exosomes could promote polarization of macrophages through STAT3 signaling pathway. To confirm that, the phosphorylation of STAT3 in macrophages treated with the exosomes (100 μg/ml) were determined. Activation STAT3 phosphorylation were observed in macrophages when they were incubated with Skov3-derived and A2780-derived exosomes (Figure 3B and Figure 3C). Moreover, STAT3 inhibitor Stattic treated macrophages for 2 hours prior to stimulate with the exosomes and explored whether the STAT3 pathway is essential for EOC-derived exosomes-modulated macrophages polarization. Western blot revealed pretreatment with Stattic impaired the effect of Skov3-derived exosomes on macrophages polarization (Figure 3D), implying STAT3 pathway played an important role in M2 polarization of macrophages.

SOCS3 is a negative regulator of STAT3 pathway [29] and has been shown to modulate macrophages polarization. Figure 3A–3C found the expression of SOCS3 was inhibited when treated with EOC-derived exosomes. These findings indicate that exosomes secreted from EOC cells might induce macrophage transformation to a M2 phenotype with the involvement of the SOCS3/STAT3 signaling pathway.

### MiR-222-3p expression in serum-derived exosomes in EOC patients

**Figure 4:**
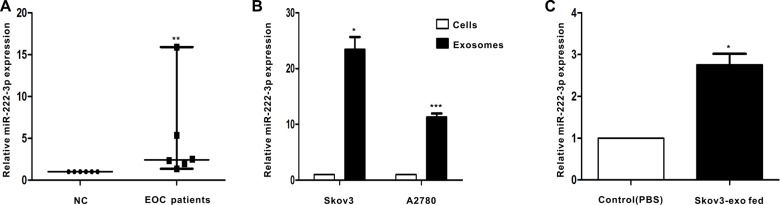
EOC patients have higher level of miR-222-3p in serum-derived exosomes than healthy individuals Besides, miR-222-3p is richer in EOC-derived exosomes and can be transferred to macrophages via the exosomes. (**A**) MicroRNAs were abstracted in serum-derived exosomes from 6 EOC patients and from 6 healthy individuals. MiR-222-3p expression was detected by qRT-PCR. Data are shown as median ± range, *n* = 6 independent experiments. **p < 0.01 compared to healthy individuals group. (**B**) The miR-222-3p level was analyzed by qRT-PCR between EOC cells and EOC-derived exosomes. In (B), *p < 0.05 compared with the Skov3 cells and ***p < 0.001 compared with the A2780 cells. (**C**) Macrophages treated with PBS or Skov3-derived exosomes for 24 hours. RNA was obtained from macrophages and the expression of miR-222-3p was performed by qRT-PCR. Data are shown as mean ± SEM, *n* = 3 independent experiments. In (B),*p < 0.05 compared with the Skov3 cells and ***p < 0.001 compared with the A2780 cells.

We next explored the level of miR-222-3p that was stored in serum-derived exosomes between healthy individuals and EOC patients (*n* = 6). Exosomal miRNAs were obtained, and qRT-PCR for miR-222-3p was performed. The expression of exosomal miR-222-3p isolated from serum in the EOC group was significantly increased compared with that in the control group (Figure 4A).

### MiR-222-3p is enriched in EOC-derived exosomes and can be transferred through the exosomes

Because exosomes contain bioactive molecules, they are involved in intercellular communication. Thus, the miR-222-3p transcript in EOC-derived exosomes was explored. The results indicated the expression of miR-222-3p in EOC-derived exosomes was much higher than that in parental cells (Figure 4B). Next, we used macrophages with Skov3-derived exosomes to determine whether the exosomes can deliver a functional transgene to macrophages. The data showed macrophages treated with exosomes expressed higher miR-222-3p than untreated macrophages (Figure 4C).

### MiR-222-3p promotes macrophage polarization and differentiation to M2 phenotypes *in vitro* and *vivo*, which can enhance the progression of EOC both

**Figure 5:**
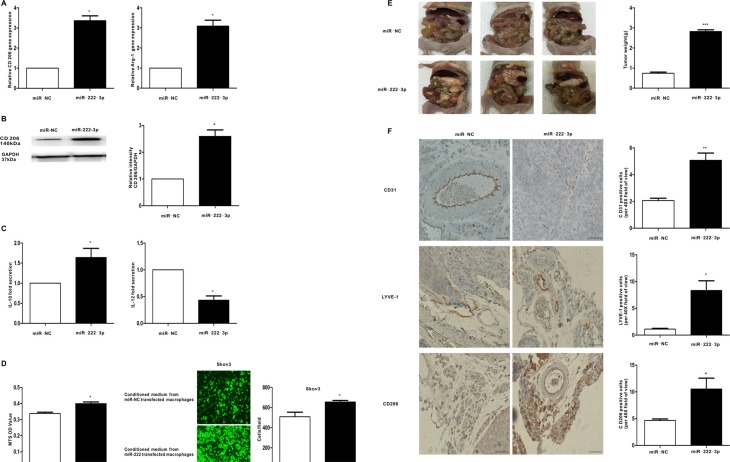
MiR-222-3p promotes M2 phenotype polarization of macrophages *in vitro* and *vivo*, which can enhance growth and metastasis of EOC cells Macrophages were transfected with the miR-222-3p mimic and compared with a negative control. (**A**) After 24 hours, qRT-PCR was applied using primers for CD206 and Arg-1. (**B**) After 48 hours, the expression of M2 surface marker CD206 was detected by western blot. (**C**) Cytokine levels in the media of macrophages. (**D**) Proliferation and migration capacity of the human EOC cell line Skov3 which incubated with the supernatants of macrophages treated with miR-222-3p or miR-negative control was performed using MTS and Transwell assay. (**E**) Images of appearance of tumour deposits at necropsy of miR-222-3p group compared with miR-NC group *in vivo*. (**F**) Representative images of CD31, LYVE-1 and CD206 immunostaining are shown in tumor tissues of miR-222-3p group or miR-NC group; scale bar = 50 μm. CD31 positive ‘hotspots’, LYVE-1 positive ‘hotspots’ and CD206-positive cells were calculated as number of positive cells. Data are shown as mean ± SEM, *n* = 3 independent experiments; *p < 0.05, **p < 0.01, and ***p < 0.001 compared with the miR-NC treatments.

To demonstrate the function of miR-222-3p in the polarization of macrophages in the tumor microenvironment, miR-222-3p mimics or the miR-negative control were transfected into macrophages, and M2 markers were detected *in vitro*. Compared with the negative control, M2 macrophage markers, including CD206 and Arg-1, were induced when macrophages were transfected with the miR-222-3p mimic (Figure 5A and Figure 5B). Moreover, supernatants from macrophages transfected with the miR-222-3p mimic or the miR-negative control were collected, and cytokine expression was detected by ELISA. In this case, the level of M2-type cytokine IL-10 increased and that of M1-type cytokine IL-12 decreased (Figure 5C). Furthermore, we explored whether miR-222-3p treated macrophages could affect the proliferation and migration of ovarian cancer cells. The conditioned medium from miR-222-3p mimics or the miR-negative control transfected macrophages was collected and provided for MTS and Transwell assay. The results indicated the conditioned medium from the miR-222-3p mimic strongly enhanced the proliferation and migration capacity of Skov3 ovarian cancer cells (Figure 5D), implying the transfection of miR-222-3p into macrophages promoted the growth and metastasis of ovarian cancer cells.

To further assess the function of miR-222-3p in transforming M1-M2 phenotype of macrophages, which stimulate the progression of EOC *in vivo*, Skov3 cells mixed with the conditioned macrophages were injected intraperitoneally into the mice. As shown in Figure 5E, compared with the control, tumors in the miR-222-3p group had a 72.79% increase in tumor weight (*P* < 0.001). Moreover, the cancer tissue expressions of CD31, LYVE-1 and CD206 were detected by IHC. Consistent with *in vitro* findings, proportion of CD206-positive cells in tumor tissue was increased in miR-222-3p group (Figure 5F, upper 3 panels). Besides, tumors from miR-222-3p group displayed more microvessels or lymphatic vessels compared with the tumors from miR-NC group, as shown by CD31 and LYVE-1 IHC (Figure 5F). Together, these results demonstrate miR-222-3p upregulated M2 macrophage polarization, which promoted the progression of cancer.

### MiR-222-3p suppresses SOCS3 expression and activates its related pathway in macrophages

**Figure 6:**
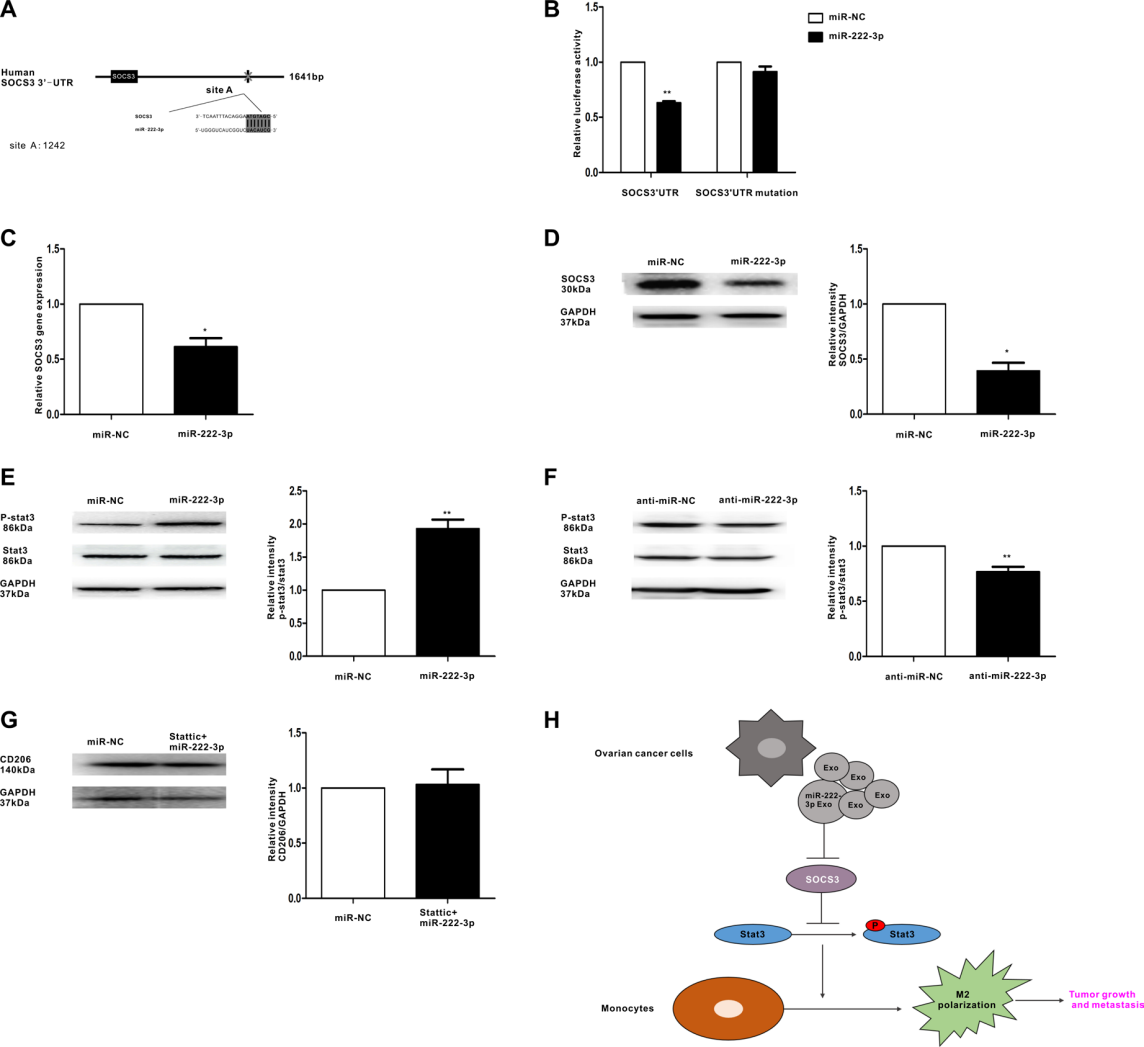
MiR-222-3p downregulates SOCS3 expression and activates STAT3 signaling pathways in macrophages (**A**) Binding sites of miR-222-3p in the 3′UTR of the SOCS3 gene according to bioinformatic analysis. (**B**) HEK293T cells were co-transfected with the 3′UTR luciferase report plasmid and miR-222-3p mimics or miR-negative control. Then, luciferase activity was measured 40 hours later. Data are presented as relative luciferase activity. (**C**) Expression of SOCS3 gene in macrophages that were transfected with miR-222-3p or miR-negative control, as performed by qRT-PCR. (**D**) A representative immunoblot of SOCS3 in macrophages which were transfected with miR-222-3p or miR-negative control. (**E**, **F**) The expressions of phosphorylated (p-) STAT3 and total STAT3 in macrophages which were transfected with miR-222-3p mimic and inhibitor were detected by western blot. (**G**) Macrophages were pretreated with STAT3 inhibitor stattic before transfecting with miR-222-3p mimic or miR-negative control. Expression of M2 marker CD206 was detected by western blot. Data are shown as mean ± SEM, *n* = 3 independent experiments; *p < 0.05 and **p < 0.01 compared with the miR-NC treatments. (**H**) Model for the role of miR-222-3p which was enriched in ovarian cancer cells derived-exosomes in the regulation of macrophage polarization in tumor microenvironment.

The TargetScan software predicted SOCS3 may be the target gene of miR-222-3p (Figure 6A). To confirm this prediction, a luciferase assay was performed. The data showed that miR-222-3p decreased the luciferase activity of Luc-SOCS3-3′ UTR and had a minimal effect on the negative control (Figure 6B). Moreover, SOCS3 expression in macrophages with miR-222-3p overexpression was examined. The results showed the expression of SOCS3 was significantly decreased when macrophages were transfected with miR-222-3p (Figure 6C and Figure 6D). Furthermore, to demonstrate the function of miR-222-3p in the STAT3 pathway, in which SOCS3 acts as the negative regulator, we further synthesized both the miR-222-3p mimic and the inhibitor and then transfected them into macrophages. In this experiment, the STAT3 pathway was either activated (Figure 6E) or inhibited (Figure 6F). Pretreatment with STAT3 inhibitor Stattic could diminish the effect of miR-222-3p on polarization of macrophages (Figure 6G). Overall, these results suggest miR-222-3p might play a role in the polarization of M2 macrophages through the SOCS3/STAT3 pathway.

## DISCUSSION

In this study, we show cellular and molecular phenomena and mechanisms involve EOC-derived exosomes inducing the activation of macrophages to a TAM-like phenotype, which may promote progression of tumors.

The exchange of cellular contents between cells plays an important role in regulating cell physiology function and can be mediated by recently found exosomes. Breast cancer-derived exosomes could regulate the pro-inflammatory activity of macrophages [30]. We first demonstrated that exosomes derived from EOC can be taken up by macrophages (Figure 1C) and are capable of inducing macrophage polarization to TAM-like phenotypes *in vitro* (Figure 2A–2C). The proliferation and migration capacity of Skov-3 could be intensified when cultured with the conditioned medium of the exosomes-treated macrophages (Figure 2D). Collectively, it is possible that EOC-derived exosomes have the ability to promote M2 polarization of macrophages correlated with the immunosuppressive phenotype, exhibited as CD206^high^Arg-1^high^MCP-1^low^IL-10^high^IL-12^low^ [31], which promoting the proliferation and migration capacity of ovarian cancer cells. *In vivo* findings suggest EOC-derived exosomes can promote polarization of TAMs, which may increase angiogenesis and lymphangiogenesis in the microenvironment (Figure 2E and Figure 2F). Thus, target the exosomes from cancer could be a promising treatment in the future.

Exosomes produced by tumor cells existed in both ascites and serum of ovarian cancer patients [16, 32] and may be linked to tumor progression. In this study, we found exosomes released from ovarian cancer cells may exist in serum. Additionally, the expression of miR-222-3p contained in exosome fractions from the serum appears to be differentiated between patients and healthy individuals (Figure 4A), indicating it can be considered a marker of cancer occurrence.

Several studies have demonstrated the existence and biological function of miRNA in exosomes [33, 34]. Here, we demonstrated miR-222-3p transcripts are significantly richer in exosomes than in their cells of origin (Figure 4B). Additionally, miR-222-3p could be transferred between ovarian cancer cells and macrophages through exosomes secreted from tumor cells (Figure 4C).

To date, miR-125, miR-155, miR-378, miR-9, miR-21, miR-146, miR-147, and miR-187 have been reported in the control of macrophage activation [9]. Our findings indicate miR-222-3p can markedly induce the M2 polarization of macrophages exhibited as CD206^high^Arg-1^high^IL-10^high^IL-12^low^ (Figure 5A–5C). In addition, the proliferation and migration capacity of the human EOC cell line Skov3 was obviously facilitated when macrophages overexpressed miR-222-3p (Figure 5D). Besides, we found miR-222-3p not only increased the population of M2 macrophages but also promoted angiogenesis and lymphangiogenesis in a tumor environment *in vivo* (Figure 5E and Figure 5F). These results, together with the above findings, suggest ovarian cancer may promote the phenotype of TAMs through transferring miR-222-3p via the secretion of abundant exosomes.

STAT3 activation is essential for the differentiation and polarization of M2 macrophages [35, 36] and SOCS3 inhibits the activation of the STAT3 pathway. Our results indicate EOC-derived exosomes can inhibit the expression of SOCS3 in macrophages and then initiate activation of the STAT3 pathway (Figure 3A–3C). MiRNAs can bind the 3′UTR location of genes, which inhibits gene expression and then degrades the mRNA [37]. Our data show miR-222-3p can target the 3′ UTR of the SOCS3 gene and inhibit its expression (Figure 6B). Then, the decreased expression of SOCS3, modulated by miR-222-3p, resulted in the phosphorylation of STAT3 (Figure 6C–6F). Accordingly, these data reveal miR-222-3p plays an important role in promoting M2 macrophage polarization by regulating the SOCS3/STAT3 pathway. What's more, STAT3 pathway is important for the modulation of the exosomes or miR-222-3P on macrophages polarization (Figure 3D and Figure 6G). Therefore, ovarian cancer cells can induce the polarization of macrophages toward M2 phenotypes via miR-222-3p is enriched in EOC-derived exosomes.

In summary, our data suggest ovarian cancer cells can transfer miR-222-3p to macrophages via exosomes, modulating the phenotype of TAMs by targeting the SOCS3 with involvement in the STAT3 pathway (Figure 6H). This may be a new mechanism related to progression of ovarian cancer. Furthermore, we observed up-regulated expression of miR-222-3p of exosomes in serum from EOC patients compared with its expression in serum from normal individuals. Thus, miR-222-3p contained in ovarian cancer derived-exosomes could become a new biomarker for diagnosis and could be a potential therapeutic target for tumor intervention.

## MATERIALS AND METHODS

### Patient samples

Blood samples from 6 healthy controls and 6 pre-therapy EOC patients were collected in a vacutainer blood collection tube (BD, USA). The tubes were centrifuged at 1500 g for ten minutes; then, the supernatants were delivered to new tubes and stored at −80°C before processing. Specimens were obtained from Shanghai First Maternity and Infant Hospital, Tongji University (Shanghai, China), according to the Ethics Committee of Shanghai First Maternity and Infant Hospital. Informed consent was obtained from patients or their guardians.

### Cell culture and treatment

The human EOC cell lines Skov3, A2780 and monocytic cell line U937 were obtained from FuHeng BIO(Shanghai, China) and maintained in RPMI 1640 medium (Invitrogen, CA, USA) containing 10% FBS (Invitrogen, CA, USA). The cell culture medium was ultracentrifuged at 100,000 *g* for 20 hours to obtain exosome-depleted medium. To induce differentiation into macrophages, U937 cells (1 × 10^6^) were treated with 100 ng/ml PMA (Sigma–Aldrich, St Louis, MO, USA) and incubated for 24 hours. After treatment, the cells were washed three times with PBS.

### Exosome isolation

Total exosome isolation reagent (from cell culture medium) (Invitrogen, CA, USA) was used to extract exosomes from cell culture medium. Skov3 or A2780 cells were grown in exosome-free medium on 10-cm dishes. Cell culture medium was harvested after 3 days (30 ml) and centrifuged at 2000 × *g* for 30 minutes to remove cells and debris. Total exosome isolation reagent was added to the cell-free culture medium and then incubated at 4°C overnight. Exosomes collected via centrifugation at 10,000 × *g* for 1 hour at 4°C were resuspended in 1 mL 1× PBS and quantified using a BCA Protein Assay Kit (Pierce Biotechnology, USA).

Total exosome isolation reagent (from serum) (Invitrogen, CA, USA) was used to isolate exosomes from serum. Serum samples (500 μl) were centrifuged at 2000 × *g* for 30 minutes to remove cells and debris. Total exosome isolation reagent was added to the serum samples and incubated at 4°C for 30 minutes. Exosomes were obtained by centrifugation at 10,000 × *g* for 10 minutes and resuspended in 200 μl 1× PBS.

### Transmission electron microscopy

Exosome pellets were dissolved in PBS buffer dropped in a carbon-coated copper grid and then stained with 2% uranyl acetate. The samples were observed using a J Tecnai G2 F20 ST transmission electron microscope.

### Exosome labeling and tracking

Purified exosomes isolated from the culture medium of Skov3 cells were collected and stained with D384 (Invitrogen, CA, USA), a phospholipid membrane dye (red stain), and SytoRNA, an RNA-select stain (green stain) (Invitrogen, CA, USA). Then, excess unincorporated dye was removed from the labeled exosomes with Exosome Spin Columns (Invitrogen, CA, USA). Labeled exosomes were added to the unstained macrophages. After incubation for 2 hours at 37°C, cells were observed using a Leica DM-LB confocal microscope.

### MiRNA transfection

Macrophages were transfected with miR-222-3p mimics, inhibitors or a miR-negative control (GenePharma, Shanghai, China) using HiperFect transfection reagent (Qiagen GmbH, Hilden, Germany).

### Prediction of SOCS3 sequence that targeted by miR-222-3p

TargetScan software predicted that the 3′ UTR of SOCS3 was potentially targeted by miR-222-3p. Predicted consequential pairing of target region and miRNA is the position 1243-1250 of SOCS3 3′ UTR (5′UGACAAUUUACAGGAAUGUAGCA) and the sequence of miR-222-3p is UGGGUCAUCGGUCUACA UCGA.

### 3′ UTR luciferase assay

The 3′ untranslated region (3′ UTR) report plasmid of the SOCS3 gene was generated by cloning the 3′ UTR downstream of the luciferase open reading frame (Hanyin Biotechnology, Shanghai, China), Then, the 3′ UTR luciferase report plasmid together with the miR-222-3p mimics or the miR-negative control was transfected with Lipofectamine 3000 (Invitrogen, CA, USA) into HEK293T cells in a 24-well plate with the indicated amount of plasmid (500 ng luciferase plasmid plus 50 nm miR-222-3p mimics). A constitutively expressed Renilla luciferase was co-transfected as a normalizing control. After 40 hours of incubation, Firefly and Renilla luciferase activities were sequentially measured using the Dual-Glo Luciferase Assay system (Promega, Madison, WI, USA).

### RNA extraction and MicroRNA profiling by RT-PCR

RNA from exosomes was isolated and enriched with a Total Exosome RNA and Protein Isolation Kit (Invitrogen, CA, USA) according to the user's guide, and the total RNA of macrophages stimulated with the exosomes (100 μg/ml) after 24 hours was extracted using Trizol (Invitrogen, CA, USA). The miScript Reverse Transcription Kit and miScript SYBR Green PCR Kit (Qiagen GmbH, Hilden, Germany) were used to reverse transcribe and detect quantitative PCR of miRNAs according to the manufacturer's protocol. A forward primer (Qiagen GmbH, Hilden, Germany) was designed to detect mature hsa-miR-222-3p. The reverse primer was used was the universal primer included in the kit. Human RNU6B was used to normalize miRNA expression. The data were calculated using the 2^−ΔΔCT^ method.

### Quantitative RT-PCR

EOC-derived exosomes (100 μg/ml) or miR-222-3p mimics and miR-negative control-treated macrophages (*n* = 3) for 48 or 24 hours were collected, and cDNA was transcribed using a PrimeScript^™^ RT Reagent Kit (Perfect Real Time) (Takara, Japan). Real-time quantitative RT-PCR was performed using the StepOnePlus™ Real-Time PCR System (Invitrogen, CA, USA) to detect mRNA of CD206, Arg-1, MCP-1 and SOCS3. Amplification was performed with SYBR^®^ *Premix Ex Taq*^™^ (Tli RNaseH Plus) (Takara, Japan), and the 2^−ΔΔCT^ method was used to calculate gene expression (with b-actin as an internal reference). The primer sequences used were as follows:

CD206 primers, forward: 5′–GGGTTGCTATCAC TCTCTATGC-3′,
reverse: 5′–TTTCTTGTCTGTTGCCGTAGTT-3′;
Arg-1 primers, forward: 5′-TGGACAGACTAGGAA TTGGCA-3′,
reverse: 5′–CCAGTCCGTCAACATCAAAACT-3′;
MCP-1 primers, forward: 5′–CAGCCAGATGCA ATCAATGCC-3′,
reverse: 5′–TGGAATCCTGAACCCACTTCT-3′;
SOCS3 primers, forward: 5′–CCTGCGCCTCAAG ACCTTC-3′,
reverse: 5′–GTCACTGCGCTCCAGTAGAA-3′;
β-actin primers, forward: 5′–CCTGGCACCCAG CACAAT-3′,
reverse: 5′–GGGCCGGACTCGTCATACT-3′.

### Western blot analyses

Total protein was isolated from macrophages were treated with EOC-derived exosomes (100 μg/ml) or miR-222-3p mimics, inhibitors and the miR-negative control for 96 or 48 hours. Antibodies against Mannose Receptor (Abcam, MA, USA), SOCS3 (Abcam, MA, USA), phospho-STAT3 (Tyr705) (Cell Signaling, MA, USA), and STAT3 (Cell Signaling, MA, USA) were used.

### ELISA assay

The cell culture medium of macrophages stimulated with Skov3-derived exosomes (100 μg/ml) or miR-222-3p mimics and the miR-negative control was collected and measured using IL-10 and IL12p70 ELISA kits (Ray Biotech, USA).

### Cell migration assay

Transwell chambers (Corning Inc, USA) with 8-μm inserts were used to measure the migration of tumor cells as indicated. Skov3 (1.5 × 10^5^ cells/chamber) was plated in the top chambers in the presence of RPMI 1640 medium containing 1% FBS, and the conditioned media of differently treated macrophages were added to the bottom transwell chamber. After 20 hours, Skov3 cells were used to count the number of cells in the bottom of the wells.

### MTS proliferation assay

Skov3 cells (2 × 10^3^) were seeded into 96-well plates and cultured with the 100 μl conditioned media of differently treated macrophages for 48 hours. Then, added 20 μl MTS Solution Reagent (Promega Biosciences, CA, USA) to each well and incubated for 2 hours at 37°C, 5% CO2 atmosphere. The absorbance was recorded at 490 nm using a 96-well plate reader.

### Animal assay

The female athymic nude mice aged 4 weeks were purchased from Shanghai JiaoTong University School of Medicine (Shanghai, China) and maintained under SPF conditions. All the animal experiments were manipulated according to guidelines approved by the Shanghai Medical Experimental Animal Care Commission. Macrophages were stably transfected with miR-222-3p-lentivirus or miR-control (Hnayin biotech, Shanghai, China). Skov3 (1.5 × 10^6^) mixed with conditioned macrophages (1:3) were injected intraperitoneally into the mice. Mice were randomly chosen and divided into 4 groups (3 animals per condition) depended on the difference of macrophages combined injection with Skov3: control group (PBS treated macrophages+ Skov3), Skov3-exo group (Skov3-derived exosomes treated macrophages+ Skov3), miR-NC group (miR-control transfected macrophages+Skov3) and miR-222-3p group (miR-222-3p transfected macrophages +Skov3). On day 28, Mice were killed and tumors were harvested for immunohistochemical analyses and weighted.

### Immunohistochemistry

Tumors were fixed, embedded in paraffin and sectioned into 4-μm thick. After deparaffinization and rehydration, sections were blocked and then incubated with anti-CD31 antibody (1:50) (Abcam, MA, USA), anti-LYVE1 antibody (1:200) (Abcam, MA, USA) and anti-CD206 antibody (1:50) (Abcam, MA, USA). A biotinylated goat anti-rabbit antibody and ABC Kit were used. CD31 positive ‘hotspots’ represented blood vessels. LYVE-1 positive ‘hotspots’ represented lymphatic vessels. CD206 positive cells were considered as macrophages. Images were quantified at 40X magnification. The quantitative analysis was made on five independent fields per tumor randomly.

### Statistics

Statistical analysis was performed using SPSS 19.0 (SPSS, USA). Data were expressed as the mean ± SEM and analyzed using Student's *t*-test or the Mann–Whitney test. All experiments for cell cultures were performed independently at least three times. A *P-*value < 0.05 was considered statistically significant.
